# Innovative Rough Beer Conditioning Process Free from Diatomaceous Earth and Polyvinylpolypyrrolidone

**DOI:** 10.3390/foods9091228

**Published:** 2020-09-03

**Authors:** Alessio Cimini, Mauro Moresi

**Affiliations:** Department for Innovation in the Biological, Agrofood and Forestry Systems, University of Tuscia, 01100 Viterbo, Italy; a.cimini@unitus.it

**Keywords:** beer, Brewers Clarex^®^, ceramic hollow-fiber membrane, colloidal stability, crossflow microfiltration, dead-end filtration, *Lactobacillus brevis*, *Saccharomyces carlsbergensis*, sensory analysis

## Abstract

In large-sized breweries, rough beer clarification is still carried out using Kieselguhr filters notwithstanding their environmental and safety implications. The main aim of this work was to test an innovative rough beer clarification and stabilization process involving enzymatic treating with Brewers Clarex^®^, centrifuging, rough filtering across 1.4-μm ceramic hollow-fiber membrane at 30 °C, and fine filtering through 0.45-μm cartridge filter. When feeding an enzymatically-pretreated and centrifuged rough beer with permanent haze (H_P_) of 2 or 14 European Brewery Convention unit (EBC-U), its primary clarification under periodic CO_2_ backflushing yielded a permeate with turbidity of 1.0–1.5 EBC-U at a high permeation flux (2.173 ± 51 or 593 ± 100 L m^−2^ h^−1^), much greater than that typical of powder filters. The final beer was brilliant (H_P_ = 0.57 ± 0.08 EBC-U) with almost the same colloidal stability of the industrial control and an overall log reduction value (~5.0 for the selected beer spoilage bacterium or 7.6 for the brewing yeast) in line with the microbial effectiveness of current sterilizing membranes. It was perceived as significantly different in flavor and body from the industrial control at a probability level of 10% by a triangle sensory test, as more likely related to the several lab-scale beer-racking steps used than to the novel process itself.

## 1. Introduction

Owing to their instability [1], the many beers produced today for the global market are to be characterized by a relatively long shelf life during which their main biological, physical, colloidal, foam and flavor quality parameters are to be kept practically unchanged from production to consumption [2]. According to Fillaudeau et al. [3], the dead-end filtration with filter-aids (such as Kieselguhr or diatomaceous earth, DE) has been the standard industrial practice for more than 100 years. In the year 2001, at the Heineken brewery in Zoeterwoude (The Nederlands), about 10 m^3^ h^−1^ of rough beer started to be filtered through 0.45-μm polyethersulfone (PES) hollow-fiber (HF) modules accordingly to the so-called Norit process [4]. Since then the number of such modules installed around the world, especially in large brewing companies, has increased, even if the great majority of breweries is still using the conventional powder filters [5].

The removal of yeast and suspended particles, after cold storage upon addition of fining agents and centrifuging, asks for long processing times, while a cloudy rough beer fed to a conventional powder filter lessens its turbidity to less than 0.5 European Brewery Convention unit (EBC-U) in few seconds [6]. High throughput capacity and filtration efficiency, as well as low operating costs, are the main advantages of DE filtration, even if the numerous environmental, sanitary, and economical issues have not been so far sufficient to make this technology obsolete [3]. Firstly, DE is a natural and finite resource [3]; secondly, it is classified as ‘hazardous waste’ before and after filtration by the World Health Organization as the crystalline silica is a cause of lung disease [3], and is responsible for arsenic contamination of beer [7]; and thirdly, the average DE sludge disposal cost into agriculture is about €170 Mg^−1^ [3].

The main critical aspects of the current beer conditioning processes concern, from one side, the removal of haze forming materials at the highest flow rates to keep lager beers brilliant even at the cold serving temperatures, and, from the other side, the microbial stability of beer.

During the cold fermentation of wort and storage of rough beer at low temperatures (−1 to −2 °C), several cross-linking molecular interactions among soluble proteins and polyphenols may occur. The removal of the resulting complexes asks for a clarification stage. Generally, the greater the proline concentration, the greater the binding affinity of proteins for polyphenols is [8]. Basically, any stabilization treatment suggested so far or currently used to prolong the colloidal stability of beer is directed to remove just a single haze precursor or both of them [8,9,10] with no impoverishing effect on other compounds responsible for beer characteristics [11].

Silica gels; isinglass, copper or kettle finings; papain; and tannic acid are the main adsorbents used to minimize the haze forming proteins in beer; while polyvinylpolypyrrolidone (PVPP) and lucilite TR (i.e., a polyvinylpyrrolidone-modified silica gel not requiring pre-swelling) to reduce the polyphenol content of beer [9]. Due to the high cost of PVPP, regenerable PVPP is preferred to single-use one, especially for breweries with an annual beer output greater than 400,000 hL [12].

A number of bacterial species possesses potential beer-spoilage ability [13,14]. *Lactobacillus* spp. and *Pediococcus* spp. are the beer-spoilage bacteria most frequently detected in breweries. Independently of the microbial type, morphology, and physiology, or contamination count, microbiological stabilization of beer up to several months is usually achieved by heat pasteurization to obtain commercial sterility of 8–30 pasteurization units (PU), 1 PU being equivalent to 1-min exposure at 60 °C [14,15]. Such heat treatment is carried out by either flash pasteurization (this allowing direct aseptic packing of pasteurized beer into metal or plastic kegs), or tunnel pasteurization (where stabilized beer as filled into precleaned glass bottles or steel or aluminum cans is pasteurized) [14]. The tunnel pasteurization system is the best one to assure sterile beer, but its main disadvantages are the loss of some flavor-producing components, and high consumption of energy for beer heating (3.0 to 3.5 kWh hL^−1^) and cooling (2.5–3.3 kWh hL^−1^), and water (~30 L hL^−1^) [16]. For flash pasteurization using plate heat exchangers, the energy consumption is about one third of the energy used in tunnel pasteurization [17]. Thus, tunnel pasteurization is the most expensive method in both capital and operating costs, these being about two or five times greater than those of sterile filtration and flash pasteurization, respectively [6].

To remove suspended particles (including yeasts and bacteria) and haze forming proteins and/or polyphenols a single operation would substantially reduce not only the operating costs, but also the environmental impact of the beer conditioning techniques [12]. Crossflow microfiltration (CFMF) might be a “wished technology” for it has been successfully applied in other food sectors over a long time [18]. Unfortunately, its use in beer clarification has been so far restricted, since the average permeation flux (50–100 L m^−2^ h^−1^) is not only about a fifth of that obtainable (250–500 L m^−2^ h^−1^) with conventional DE filters [3,5,19], but also it is highly dependent on the initial turbidity of beer [20]. Moreover, even if the resulting beer permeate is microbiologically safe, it cannot undergo aseptic filling unless rough beer has been previously stabilized [12].

The aforementioned technical limits of CFMF are, in all probability, due to an incorrect implementation of such technology. The simple replacement of DE filters with CFMF units inevitably led to choose suboptimal operating conditions. Despite both these technologies operate with the same purpose, they have completely different characteristics and limits. In addition, polymeric membranes generally suffer from an average lifetime of just two years [5], and cannot be cleaned and sanitized using high chemical concentrations and temperatures, these conditions being necessary when dealing with heavy fouling liquids, such as rough beer [21]. On the contrary, these membranes are indeed appropriate for sterilizing or safety filtering (Filtrap) pre-clarified liquids, this allowing them to operate in optimal conditions with minimal fouling. On the other hand, in spite of their quite low filtration rates, ceramic membranes may operate with thick liquids and undergo very severe cleaning protocols at low pH values and high temperatures, as well as steam sterilization [18].

Viscosity is one of the physical properties affecting the performance of filtration processes and, therefore, is of great importance in the clarification of beer. Several viscosifying components (e.g., β-glucans, arabinoxylans, and dextrins) are however contributing, within proper limits, to the overall sensory quality of beer, specifically clarity, foam, and texture. High concentrations of these substances give the beer a high viscosity, this being obviously greater at low temperatures. Noticeably, there is inverse correlation between the filtration flow rate and beer viscosity [22,23]. The latter can be reduced by depolymerizing enzymatically such viscous components, using malt o barley with low β-glucans concentration, or simply increasing the filtration temperature (TF). In particular, as TF was increased from 3 °C to 40 °C, the viscosity of a pre-centrifuged rough beer, as such or permeated across a 0.8-μm ceramic hollow-fiber (HF) membrane module, reduced from about 3 to 0.9 mPas [24], while that of pure water reduced from ca. 1.62 to 0.66 mPas [25]. The contribution of the above components to beer viscosity was definitively higher in the range of 3 °C to 10 °C than in that of 30 °C to 40 °C.

Thus, to enhance the permeation flux, the CFMF of rough beer should be carried out at greater temperatures than those (0–2 °C) presently used with DE filters, and what is more the feed should have been pre-stabilized. The colloidal stability of the rough beer as submitted to enzymatic treatment with Brewers Clarex^®^ (BC), PVPP, or both pretreatments was compared [24]. The only BC treatment was able to confer almost the same (if not better) colloidal stability of the rough beer conventionally treated with PVPP. Since such an enzymatic pretreatment is generally carried out during the fermentation or maturation step, there should be no need for filtering rough beer at low temperatures.

The main aim of this work was to assess the performance and sensitivity of a novel rough beer conditioning process consisting of a primary clarification step of a rough beer enzymatically pretreated with Brewers Clarex^®^ via 1.4-μm ceramic HF membrane module at a high filtration temperature of 30 °C, and a secondary sterilization step using a 0.45-μm polyethylene terephthalate (PET) cartridge filter, in order to yield a beer ready for aseptic packaging. To this end, first, some crossflow microfiltration tests of rough beer samples with different permanent haze values were carried out at different filtration temperatures. Second, a final validation test was designed to check for the process effectiveness by assessing not only the average permeation flux, but also the chemico-physical, colloidal, microbial, and sensory quality of micro-filtered beer.

## 2. Materials and Methods

### 2.1. Raw Materials

The rough beer used in this work was produced from malted barley (60% *w*/*w*) and corn grits (40% *w*/*w*) in an Italian industrial-scale brewery using a three-step decoction system and the high-gravity brewing method [26]. Each production lot was of about 1400 hL, while the conventional filtration plant mainly consisted of a DE candle filter and a PVPP-regenerating filter with an overall throughput of about 600 hL h^−1^. It was pre-treated with a dose of 30 μL L^−1^ of the commercial enzymatic preparation Brewers Clarex^®^ (BC: DSM Food Specialties, Delft, NL, USA), this being rich in prolyl oligopeptidase (EC 3.4.21.26) of fungal origin. The resulting gluten-free rough beer was withdrawn from one of the industrial maturation tanks, collected in three 30-L stainless steel kegs, stored at (0.5 ± 0.5) °C, and used as reported below.

The same type of beer, as BC-treated and filtered across DE filters, packed in 33-cL glass bottles and tunnel pasteurized by the same brewery, was used to test the microbiological effectiveness of the novel conditioning process under study.

### 2.2. Equipment and Experimental Procedure

The same bench-top CFMF plant previously described [27,28] was used. It was equipped with a 1.4-μm ceramic HF InoCep^®^ membrane module type MM04 (Hyflux Ltd., Singapore [29]). It was composed of 40 hollow-fibers (inside diameter = 3 mm; overall length = 200 mm) and had an effective membrane surface area (A_m_) of 0.04 m^2^. The beer permeate was then dead-end filtered through a 0.45-μm PET Tandem cartridge filter (Tenco srl, Avegno, Italy) and finally aseptically packed in pre-sanitized 30-L stainless steel kegs.

Two of the kegs mentioned above were used for the following trials. Once the rough beer had been charged in 0.3-L plastic bottles, a Beckman J2-21 floor model centrifuge operating at 6000× *g* and ~4 °C for 10 min was used to remove suspended solids and thus standardize the initial turbidity (H_P_) at two levels (i.e., 2 and 15 EBC-U). After having charged the beer tank with circa 7 L of the above pre-centrifuged rough beers, a few CFMF trials were performed in the total recycle mode to assess the effects of TF and H_P_ on the permeation flux (J_V_) under the following prefixed conditions (i.e., feed superficial velocity, v_S_, of 2.5 m s^−1^; pressure at the permeate exit port, P_P_, of ~1 bar; transmembrane pressure difference, TMP, of c. 2.5 bar). As J_V_ declined up to reach nearly a constant value (its coefficient of variation being smaller than 5%) for 15–20 min, such a value was recorded as the *limiting permeation flux* (J*) [18]. Such trials were initially carried out at TF = 10.0 ± 0.5 °C. Successively, TF was increased in steps of 10 °C up to 40 °C. Two digital flow-meters with measuring ranges of 0.06–1.8 m^3^ h^−1^ and 6–180 L h^−1^ and accuracy of ±1% at the top scale were used to quantity the retentate (Q_R_) and permeate (Q_P_) flow rates, respectively. Primarily, the instantaneous permeation flux (J_V_) was calculated as:

(1) $$J_{V}\left(t\right)=\frac{Q_{P}\left(t\right)}{A_{m}}$$

while the average permeation flux (J_Va_) was estimated via the Simpson’s rule of integration between the starting and end (t_f_) times of each trial with a time increment of 1 min as:

(2) $$J_{Va}=\frac{\int_{0}^{t_{f}}J_{V}(t)dt}{t_{f}}$$

The validation CFMF trials were performed in the batch mode by recovering the permeate and recycling the retentate back to the feed tank. The rough beer contained in the aforementioned third keg was not preliminarily centrifuged, but directly micro-filtered to limit as much as possible the oxidation of polyphenols and guarantee the sensory quality of the micro-filtered beer. Actually, the first lot of approximately 8 L was directly siphoned from the keg as it was, thus obtaining a lot with an initial turbidity of ~14 EBC-U. The second lot with a low turbidity of ~2 EBC-U was recovered after the keg had been conditioned at circa 0 °C for 7 days. The CFMF conditions were set as follows: TF = 30.0 ± 0.5 °C, v_S_ = 2.5 m s^−1^, P_P_ ≈ 1 bar, TMP ≈ 2.5 bar. During such trials, periodic CO_2_ backflushing was used to restore the instantaneous J_V_ as reported in previous work [20]. To detect the microbial reduction level in the beer clarified under the process conditions mentioned above, several 33-cL glass bottles of gluten-free beer were firstly degassed using a ultrasonic water bath for 10 min; secondly, transferred into the feed tank of the CFMF bench-top plant, and then inoculated with WLP672 *Lactobacillus brevis*—Purepitch (White Labs Copenhagen, Kastrup, Denmark) and dry brewing yeast (Fermentis SafAle^™^ S-33, Société Industrielle Lesaffre, Marcq-en-Baroeul, France). An initial concentration of about 10^3^ cell mL^−1^ of *Lactobacillus brevis* was used to simulate the contamination level regarded as critical in several craft beers [30]. As concerning the brewing yeast, 6.0 g of dry yeast were pre-hydrated in 50 mL of beer and then pitched into circa 8 L of the beer undergoing CFMF. The CFMF plant was kept operating in the total recycle mode for about 30 min. After that, three 500-mL samples were withdrawn from the retentate outlet (coded R), permeate outlet (coded P), and 0.45 μm cartridge filter exit (coded CF) and collected in sterile containers by flaming over a Bunsen burner. Of such samples, two ones were used for the microbiological testing and the remaining one for the chemico-physical analyses.

Finally, the sensory tests were carried out on enzymatically-treated and centrifuged rough beer, as permeated across the 1.4-μm ceramic HF membrane, filtered through the 0.45-μm PET cartridge filter, aseptically collected in pre-sanitized stainless steel kegs, stored at −1 °C, and saturated with CO_2_ at a pressure of 2 bar for longer than 7 days.

Preliminarily to each CFMF trial, the membrane module was cleaned using the multi-step procedure previously described [28].

### 2.3. Analytical Methods

All beer samples collected were analyzed using the European reference methods for breweries by the European Brewery Convention (EBC) [31]. In particular, the density (ρ_B_), viscosity (η_B_), and ethanol volumetric fraction (y_E_) of the beer samples were determined by using specific instruments provided by Anton Paar Italia Srl (Rivoli, Italy), such as DMA™ 4500 M density meter (density measurement range: 0–3 kg L^−1^ at 20 °C, accuracy: 0.00005 kg L^−1^; resolution: 0.00001 kg L^−1^), rotating ball viscometer Lovis 2000 M/ME, and Alcolyzer Wine M/ME (measuring range: 0–20% *v/v*, repeatability standard deviation: ± 0.01% *v/v*). The turbidity or permanent haze (H_P_), as well as chill haze (H*_C_*), color (C), total phenol content (TP), sensitive protein content (SP) and alcohol chill haze or Chapon test (ACH) were determined in accordance with the EBC methods no. 9.29, 9.6, 9.11, 9.40, and 9.41, respectively [31]. The beer foam half-life (t_½_) was estimated by using the improved Rudin-based beer head retention meter, described previously [32].

Analytical grade reagents were used for all the chemical analyses.

Bacterial (N_B_) and yeast (N_Y_) counts were determined according to the norm ISO no. 15214: 1998 [33] and EBC method no. 3.1.1.1 [34], respectively. In this way, it was possible to estimate the log reduction value (LRV) as the decimal logarithm of the ratio between the numbers of bacterial or yeast cells at the retentate outlet and permeate or cartridge filter exit.

### 2.4. Sensory Analysis

A triangle test was performed in accordance with the EBC method no. 13.7 (Sensory Analysis: Triangle Test) [31] to assess whether the micro-filtered beer (MFB), as obtained from the conditioning process proposed in this work, was significantly different from the same beer (STD) conventionally conditioned (that is, clarified, stabilized using DE and PVPP filtration, bottled and tunnel pasteurized). By assuming 10% risk of concluding that a significance difference between MFB and STD existed when, in reality, there was not one (α ≤ 0.1), 10% risk of concluding that no significant difference between MFB and STD existed when, in reality, there was one (β ≤ 0.1), and a 50% proportion of distinguishers, at least 15 assessors were to be used (see Table 1 of Section 13.7 [31]). By accounting for the six possible combinations (i.e., 112, 121, 122, 212, 221, 222), 18 assessors of whom 7 females and 11 males between 28 and 50 years of age were invited to balance the order of sample presentation. The panelists were trained professionals recruited from a group of selected employees of the industrial brewery, each one following a continuous training in accordance with the EBC method no. 13.4 (Sensory Analysis: Selection and Training of Assessors) [31] and weekly beer analysis programs. Each evaluation was performed in individual booths of the brewery sensory laboratory, where each panelist received three samples, each one of 200 cm^3^ at about 4 °C, in 3-digit coded capped glass cups to identify, which sample was different and remark the most relevant attributes perceived.

### 2.5. Statistical Analysis of Data

The chemico-physical properties of each beer sample were measured three times. The CFMF trials were repeated two times. Results are the means of replicated experiments ± standard deviation. Data were analyzed by one-way ANOVA and post-hoc Tukey test at a significance level of 0.05, using the SigmaStat 3 software (Jandel Corp, San Rafael, CA, USA).

## 3. Results and Discussion

### 3.1. Permeation Flux vs. Filtration Temperature

**Figure 1:**
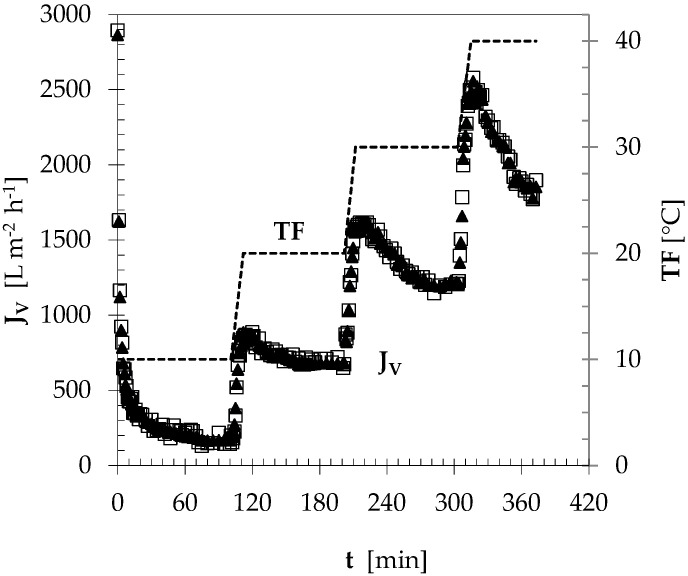
Effect of the filtration temperature (TF: - -) on the time course of the permeation flux (J_V_: ☐, ∆) of pre-centrifuged rough beer in two crossflow microfiltration (CFMF) trials performed in the total recycle mode as reported in the text.

By operating the bench-top CFMF plant in the total recycle mode at v_S_ = 2.5 m s^−1^, P_P_ = 1 bar, TMP = 2.4 ± 0.1 bar, and TF increasing from 10 °C to 40 °C by steps of 10 °C, the instantaneous beer permeation flux (J_V_) exhibited the time course shown in Figure 1 in the case of pre-centrifuged samples of rough beer with turbidity of ~2 EBC-U.

At TF = 10 °C, J_V_ was initially equal to 1600–3000 L m^−2^ h^−1^, but abruptly declined to reach the limiting value (J*). The instantaneous J_V_ values were averaged over an overall time interval of about 100 min or during the latest 15–20 min. As shown in Table 1, J_Va_ was equal to 297 L m^−2^ h^−1^, and J* to 163 L m^−2^ h^−1^. By increasing the filtration temperature to 20 °C, J_V_ exhibited a prompt increase, followed by another decline. Such a trend was confirmed as TF was increased to 30 °C, and then to 40 °C. As TF was increased from 10 °C and 40 °C, J_Va_ and J* improved from 297 to 2090 L m^−2^ h^−1^, and from 163 to 1855 L m^−2^ h^−1^, respectively (Table 1). Thus, at TF ≥ 20 °C the average permeation flux across 1.4-µm ceramic HF membrane was remarkably greater than that (250 to 500 L m^−2^ h^−1^) achievable with conventional DE filters [3,5,19]. Such results were practically in line with those previously observed when filtering a pure malt rough beer through the same membrane module under the identical operating conditions [24]. The influence of CFMF at low temperatures of 2–3 °C on ceramic membrane fouling and beer quality was largely assessed only in the case of tubular ceramic membranes with nominal porosity in the range of 0.2–1.3 μm [35,36,37,38]. Maximum permeation flux values of 20–30 L m^−2^ h^−1^ were observed, this confirming that rough beer should be filtered at higher temperatures to obtain a flux of industrial relevance [37].

### 3.2. Permeation Flux vs. Feed Turbidity

**Figure 2:**
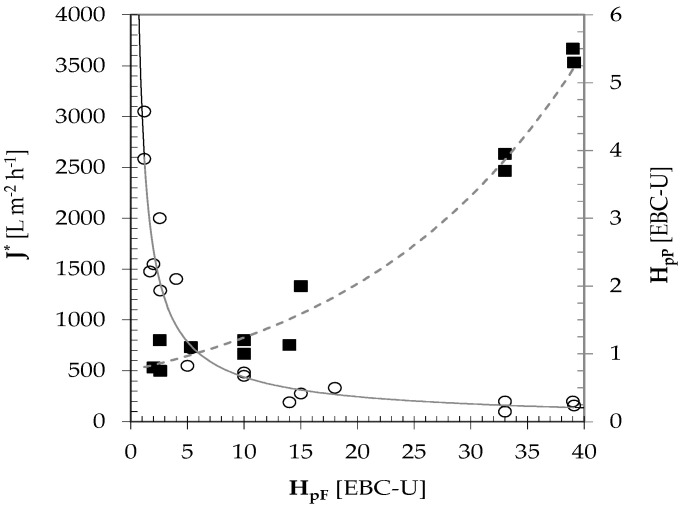
Effect of rough beer turbidity (H_pF_) on the limiting permeation flux (J*: ◯) and turbidity (H_pP_: ■) of the permeate collected from the CFMF plant operating as reported in the text. The continuous and broken lines represent the least square regression lines.

Figure 2 shows the limiting permeation flux (J*) and permeate turbidity (H_pP_) as a function of the permanent haze (H_pF_) of the rough beer fed to the CFMF bench-top plant under the aforementioned operating conditions. The paramount effect of the feed turbidity on J* was thus acknowledged. For H_pF_ increasing from 1 EBC-U to 2 EBC-U, J* decreased from 3000 L m^−2^ h^−1^ to 2.000 L m^−2^ h^−1^; while for H_pF_ increasing from 5 EBC-U to 10 EBC-U, J* exhibited a mean value of 550 ± 120 L m^−2^ h^−1^. The observed J* values were by far higher than or near to the typical fluxes (250–500 L m^−2^ h^−1^) achieved with powder filters [3,5,19]. As H_pF_ increased from 10 EBC-U to 40 EBC-U, J* levelled to 164 ± 48 L m^−2^ h^−1^. By the other side, the greater the feed turbidity the greater the permanent haze (H_pP_) of the beer permeated will be. For H_pF_ ranging from 1 EBC-U to 15 EBC-U, H_pP_ increased from 0.7 EBC-U to 1.3 EBC-U. When the feed turbidity was as high as 40 EBC-U, H_pP_ increased up to ~5.5 EBC-U.

### 3.3. Sensitivity of the Permeation Flux to Feed Turbidity and Crossflow Velocity 

When dealing with a rough beer of low turbidity (~1.5 EBC-U), the average permeation flux at 30 °C resulted to be quite insensitive to the crossflow velocity (v_S_), as shown in Table 2. In the circumstances, it was sufficient to recirculate as little as 3.8 L of retentate per each L of permeate withdrawn, probably because of the development of a thin cake over the membrane surface. This involved a crossflow velocity of just 0.5 m s^−1^, while a recirculation ratio (R/P) as high as 22 L L^−1^ ensured a five times greater superficial velocity in each hollow fiber membrane. In this way, it would be possible to minimize not only the pumping energy consumption, but also the mechanical breakup of larger protein-polyphenol complexes, this generally resulting in finer haze particles. On the contrary, when operating with a highly turbid feed (~33 EBC-U) the effect of the recirculation ratio (R/P) on the average permeation flow was indisputable (Table 2). As v_S_ was decreased from 2.5 to 0.5 m s^−1^, J_Va_ reduced from 350 to 50 L m^−2^ h^−1^ in spite of using recirculation ratios as high as 181–260 L L^−1^.

Based on these results, it would be possible to suggest starting the clarification of rough beer at low v_S_ values, such as 0.5 m s^−1^. To counteract the more or less significant reduction in J_Va_, or increase in retentate turbidity, v_S_ might be sequentially increased while keeping Transmembrane pressure difference (TMP) constant during the batch CFMF. Obviously, when using efficient centrifuges, appropriate maturation times and adjuvants to favor beer clarification, it would be in principle possible to obtain a pre-treated rough beer with low turbidity, even if this may be an optimistic condition. It is also worthwhile underlining that such procedure resulted to be much more effective than that involving a gradual increase in TMP under constant cross-flow velocity to assure an average permeation flux of 80–100 L m^−2^ h^−1^. Such a procedure was either suggested by the ceramic HF membrane module manufacturer [29] or applied to run the PES HF membrane modules at the Heineken brewery mentioned above [4].

### 3.4. Validation Testing

#### 3.4.1. Permeation Flux under Periodic CO_2_ Backflushing

**Figure 3:**
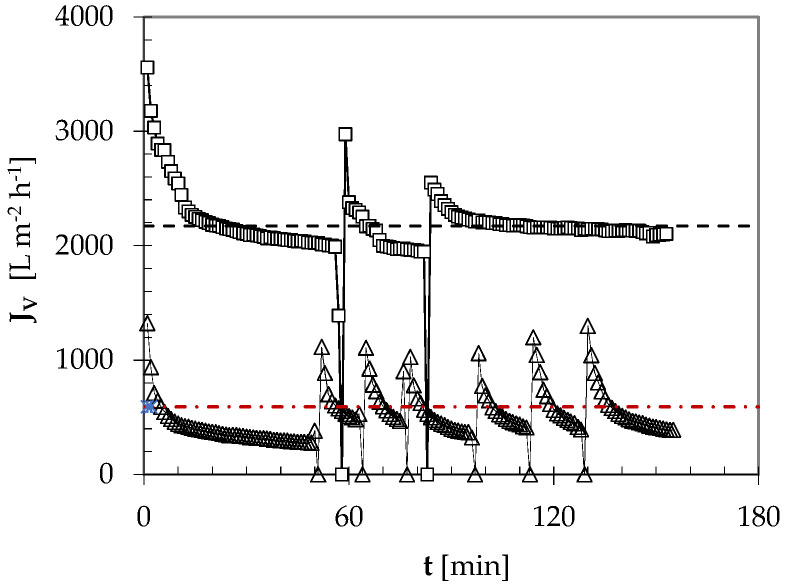
Time course of the instantaneous permeation flux (J_V_) of two rough beer samples having high (~14 EBC-U: ∆) or low (~2 EBC-U: ☐) turbidity fed to the CFMF plant under the operating conditions given in the text, and periodic CO_2_ backflushing. For all characteristics of rough beer samples, see Table 3. The average coefficient of variation for J_V_ was ± 10%. The broken horizontal lines refer to the average permeation flux achieved in any of the two trials.

The novel conditioning process was tested by feeding the CFMF plant with rough beer samples at two turbidity levels (i.e., circa 2 and 14 EBC-U). Under the aforementioned conditions (i.e., v_S_ = 2.5 m s^−1^; TF = 30 °C, P_P_ = 1 bar; TMP = 2.4 bar), the use of the lower turbid feed resulted in an initial permeation flux J_V_ of about 3500 L m^−2^ h^−1^. After circa 60 min, J_V_ lowered to ~2000 L m^−2^ h^−1^; then, periodic CO_2_ backflushing succeeded in keeping about constant the average permeation flux (2173 ± 51 L m^−2^ h^−1^), as shown in Figure 3. 

As expected, the use of the higher turbid feed led to a lower initial J_V_ value (~1300 L m^−2^ h^−1^). It, however, dropped to 280 L m^−2^ h^−1^ in less than 50 min (Figure 3). Intermittent CO_2_ backflushing yielded an average permeation flux of 593 ± 100 L m^−2^ h^−1^ for as long as 3 h. In both trials, the permeate exiting from the 1.4-μm ceramic HF membrane module at 30 °C exhibited a permanent haze (H_pP_) of 1.0–1.5 EBC-U. Fine filtering through a 0.45-µm PET cartridge filter caused no clogging problems, and produced a brilliant beer with a permanent haze around 0.6 EBC-U (Table 3). It was then aseptically collected in a keg to be used for the chemico-physical and sensory analyses.

#### 3.4.2. Chemico-Physical Characteristics and Colloidal Stability of Permeated Beer

As shown in Table 3, the density (ρ_B_) and viscosity (η_B_) at 20 °C, and real extract (RE) of the stabilized and clarified beer (MFB) resulting from the conditioning process proposed here were not significantly different from those of the same beer (STD) conventionally conditioned (that is, BC-treated, DE and PVPP filtered, bottled, and tunnel pasteurized).

The sample MFB presented a slightly higher turbidity value than the control one (0.57 ± 0.08 vs. 0.50 ± 0.06 EBC-U), less color (6.9 ± 0.1 vs. 7.3 ± 0.1 EBC-U) and smaller foam retention time (89 ± 2 vs. 96 ± 2 s). This was, in all probability, due to the retention of some beer components on the membrane surface. However, such an effect is certainly of minor importance, since it can be quite easily overcome by appropriate changes in the recipe formulation, as well as using some additive to enhance foam and color retention.

The main parameters measuring the colloidal stability of MFB and STD samples are also listed in Table 3.

Since the micro-filtered beer MFB was not treated with PVPP, its total polyphenol content (143 ± 5 mg L^−1^) was obviously higher than that (99 ± 3 mg L^−1^) assessed in the STD sample. In the sample MFB, the chill haze development (ΔH_CPH_) was found to be approximately 0.8 ± 0.3 EBC-U, about 40% less than in the control. By measuring the increase in the permanent haze of both samples 40 min after adding tannic acid, or ethanol and chilling to −5 °C, the MFB sample displayed just slightly higher ΔH_SP_ and ΔH_E_ values than those observed in the STD sample (Table 3).

#### 3.4.3. Microbiological Characterization

To assess the effectiveness of microbial retention by CFMF membranes, the same type of beer (that is BC-treated, DE filtered, packed in 33-cL glass bottles and tunnel pasteurized) was inoculated with the two target microorganisms generally present in rough beers.

Table 4 shows the number of bacterial (N_B_) and yeast (N_Y_) cells counted in the beer samples aseptically collected at the retentate (R), permeate (P) and cartridge filter (CF) exit ports of the CFMF bench-top plant used here, as well as the corresponding logarithmic reduction values (LRV).

In brief, the 1.4-μm ceramic HF membrane module allowed 3.8–4.0 or 4.2–4.5 log reduction of the initial cell numbers of the selected beer and brewery-related spoilage bacterium or yeast, respectively. Final filtering of permeated beer through a 0.45-μm cartridge filter resulted in an overall log reduction value of 4.4–5.0 or 7.6–7.7 for the aforementioned selected microorganisms. As expected, these LRVs for the target bacterium were smaller than those obtained for the target yeast of choice.

Despite an LRV value of 5–7 is normally believed acceptable for food and beverage applications, the EBC Manual of Good Practice [39] recommends a minimum commercially sterility of 15 or 20 PU for Pilsner and lager beers or ale and stout, respectively, this indicating a >8.7 log reduction in the cell numbers of the selected organisms [40]. Such EBC recommendations refer to the studies carried out as long ago as 1951 [41]. In all probability, the high heat loads involved during beer pasteurization result in damage to aroma and flavor compounds [42]. As pointed out by Rachon et al. [40], the EBC guidelines were composed more than 20 years ago and hygiene in breweries has by far improved since then. Thus, lower PUs and, consequently, lower LRVs, as those achieved with current membrane filters, should be today sufficient to achieve microbial stability in beer [40].

#### 3.4.4. Sensory Analysis

Table 5 shows the results of the triangular test designed to compare three coded samples, two of which being identical. Sample 1 was withdrawn from commercial 0.33-cL beer bottles, while sample 2 from the keg containing the same type of beer stabilized, clarified, and cartridge filtered using the conditioning process under study.

As shown by the randomized sample plan, ten assessors out of 18 correctly identified the odd sample. Table 3 of Section 13.7 [31] specifies that a minimum of 10 correct responses is necessary to conclude that a significant difference between samples 1 and 2 existed at confidence level of 90% (α risk = 10%).

As concerning the attributes perceived, the MFB sample was retained more oxidized with a lower level of bitterness, carbonation, and caramel flavor, and even with phenolic and wine off flavors. Such oxidation off flavor was in all probability more closed related the numerous lab-scale beer racking steps used than to the novel process itself, or to the bench-top plant size used, even if the filtration process was carried out under CO_2_ atmosphere.

## 4. Conclusions

In this work, the performance of a novel DE- and PVPP-free beer conditioning process operating at 30 °C was assessed. Provided that centrifuging a rough beer enzymatically pretreated with Brewer Clarex^®^ allowed its permanent haze to be reduced to about 2 EBC-U or 14 EBC-U, its primary clarification across 1.4-μm ceramic HF membrane module under periodic CO_2_ backflushing yielded a permeate with turbidity of 1.0–1.5 EBC-U at an average permeation flux of 2173 ± 51 or 593 ± 100 L m^−2^ h^−1^, respectively. Final filtering through a 0.45-µm cartridge filter not only proceeded with no clogging problems, but also gave rise to a brilliant, colloidally stable, and microbiologically stabilized beer. In fact, its permanent haze equaled to 0.57 ± 0.08 EBC-U; its responsiveness to chilling, sensible proteins and alcohol chill haze was almost in line with that of the industrial control, and its microbial stability was characterized by an overall log reduction value of about 5.0 for the selected beer spoilage bacterium or 7.6 for the brewing yeast, this being generally regarded as acceptable for food and beverage applications. Finally, a significant difference in flavor and body between the control and permeated beer samples at a probability level of 10% was revealed by a triangle sensory test, which was likely more related to the several lab-scale beer-racking steps used than to the novel process itself.

Further testing of this novel conditioning process on the pilot-plant scale is thus needed.

## Figures and Tables

**Table 1:** Effect of temperature (TF) on the mean values and standard deviations of the average (J_Va_) and limiting (J*) permeation fluxes across 1.4-µm ceramic hollow-fiber (HF) membrane module when using Brewers Clarex^®^ (BC)-treated and centrifuged rough beer with H_P_ ≈ 2 European Brewery Convention unit (EBC-U).

TF [°C]	J_Va_ [L m^−2^ h^−1^]	J* [L m^−2^ h^−1^]
10	297 ± 2	163 ± 21
20	709 ± 5	687 ± 19
30	1331 ± 1	1204 ± 22
40	2090 ± 6	1855 ± 46

**Table 2:** Effect of feed permanent haze (H_pF_) and superficial velocity (v_s_), or feed flow rate (F), on the permeate flow rate (P), average permeation flux (J_Va_), and recirculation ratio (R/P) when operating at 30 °C with 1.4-μm ceramic HF membrane module under the operating conditions given in the text.

H_pF_ [EBC-U]	v_S_ [m s^−1^]	F [L h^−1^]	P ^1^ [L h^−1^]	J_Va_ [L m^−2^ h^−1^]	R/P [L L^−1^]
1.5	2.5	2545	110 ^a^	2750	22.1
1.5	1.0	1018	114 ^a^	2850	7.9
1.5	0.5	509	105 ^a^	2625	3.8
33	2.5	2545	14 ^b^	350	180.8
33	1.0	1018	4 ^c^	100	253.5
33	0.5	509	2 ^d^	50	260.5

^1^ The average coefficient of variation for P was ± 10%. Different lowercase Latin letters indicate statistically significant difference among the permeate flow rates at the probability level of 0.05.

**Table 3:** Main chemico-physical (density, ρ_B_, and viscosity, η_B_, at 20 °C; real extract, RE; color, C; permanent haze, H_p_; total polyphenol content, TP; alcohol titer, y_E_; beer foam half-life, t_½_) and colloidal (haze increase resulting from chilling, ΔH_CPH_, and tannic acid, ΔH_SP_, or alcohol, ΔH_E_, addition,) characteristics of the beer samples micro-filtered beer (MFB) and significantly different from the same beer (STD) examined in this work.

Beer Sample	ρ_B_ [kg L^−1^]	η_B_ [mPa s]	RE [° P]	C [EBC-U]	H_p_ [EBC-U]	TP [mg L^−1^]	y_E_ [% *v/v*]	t_½_ [s]	ΔH_CPH_ [EBC-U]	ΔH_SP_ [EBC-U]	ΔH_E_ [EBC-U]
MFB	1.004 ± 0.000 ^a^	1.408 ± 0.008 ^a^	1.53 ± 0.01 ^a^	6.9 ± 0.1 ^a^	0.57 ± 0.08 ^a^	143 ± 5 ^a^	4.62 ± 0.04 ^a^	89 ± 2 ^a^	0.8 ± 0.3 ^a^	4.7 ± 0.3 ^a^	4.0 ± 0.3 ^a^
STD	1.004 ± 0.000 ^a^	1.415 ± 0.012 ^a^	1.56 ± 0.03 ^a^	7.3 ± 0.1 ^b^	0.50 ± 0.06 ^b^	99 ±3 ^b^	4.66 ± 0.06 ^a^	96 ± 2 ^b^	1.3 ± 0.2 ^b^	4.1 ± 0.2 ^b^	3.0 ± 0.6 ^b^

Different lowercase Latin letters indicate statistically significant difference among the parameter means at the probability level of 0.05.

**Table 4:** Main results of the microbiological analyses: number of bacterial (N_B_) or yeast (N_Y_) cells in samples aseptically collected at the retentate (R), permeate (P), and cartridge filter (CF) exit ports of the CFMF bench-top plant used in this work.

Target Microorganism	*L. brevis*				*S. carlsbergensis*			
Test no.	1		2		1		2	
Sample	N_B_^1^	LRV	N_B_^1^	LRV	N_Y_^1^	LRV	N_Y_^1^	LRV
R	1.05 × 10^3^	0.0	1.10 × 10^3^	0.00	1.70 × 10^6^	0.00	1.70 × 10^6^	0.00
P	1.60 × 10^−1^	3.82	1.10 × 10^−1^	4.00	1.09 × 10^2^	4.19	5.60 × 10^1^	4.48
CF	4.20 × 10^−2^	4.40	1.00 × 10^−2^	5.04	4.25 × 10^−2^	7.60	3.60 × 10^−2^	7.67

^1^ [CFU mL^−1^].

**Table 5:** Main results of the triangular test: randomized sample presentation order in each booth, different sample identified (DSI), and comments by each panelist.

Booth no.	Key	DSI	Panelist’s Comments
1	212	1	1: more caramel, oxidized flavored, and less crispy
2	212	1	2: less clean and astringent, but heavier in body
3	112		
4	221	1	1: bitter and more carbonated
5	211	2	2: oxidized flavor
6	112		
7	212		
8	221		
9	122		
10	211	2	2: phenolic flavor
11	121	2	2: less bitter and more ester flavored
12	122	1	2: less ester flavored, grainy, and acetaldehyde off-flavors
13	221		
14	121		
15	122	1	1: more carbonated
16	211		
17	112	2	2: wine taste in the after taste
18	121	2	2: oxidized flavor and less carbonated

Sample 1: STD; Sample 2: MFB.
